# The impact of patient- and SBI process-level variables on six-month drinking outcomes in a level I trauma center

**DOI:** 10.1186/1940-0640-8-S1-A59

**Published:** 2013-09-04

**Authors:** Jennifer L Rogers, Laura Veach, Preston Miller, Mary Claire O’Brien

**Affiliations:** 1 Department of Counseling, Wake Forest University, Winston-Salem, NC, USA; 2 Wake Forest School of Medicine, Winston-Salem, NC, USA; 3 Department of Counseling, University of North Carolina at Charlotte, Charlotte, NC, USA

## 

Researchers have decried the lack of investigation regarding the “how and why” of brief intervention effects. Additional research is needed to explicate the processes at work during motivational interviewing brief interventions, as well as the most salient factors influencing these processes. The overall aim of the current study was to better understand the nature and possible active ingredients of the screening and brief intervention encounter. Specifically, this inquiry focused upon the effect of patient-level variables and two distinct brief motivational interviewing-based counseling interventions on six-month drinking outcomes. Three hundred and thirty-three adult participants were recruited from a Level I trauma center. Participants were randomized to one of two brief counseling intervention arms. All interventions were conducted by professional mental health counselors or graduate student counselors-in-training. Follow-up data regarding participants’ drinking patterns and well-being were collected via telephone approximately 6 months subsequent to the intervention. Data were analyzed using MANOVA and hierarchical regression. There were two primary significant findings of this study: one, that a new standardized brief counseling intervention appeared to be as efficacious as a widely-used brief intervention focusing upon quantity and frequency of drinking; and two, that patient pre-intervention AUDIT scores were a major predictor of changes in patient AUDIT scores at 6 months post-intervention in a way that was theoretically counter-intuitive: namely, that high scores (indicating possible alcohol dependence) were associated with the greatest changes at follow-up. Limitations and implications of findings are discussed. Future investigation of the construct of patient engagement is recommended.

